# Aggressive Multiple Central Giant Cell Granulomas of the Jaws

**DOI:** 10.1155/2023/5410229

**Published:** 2023-02-03

**Authors:** Farnoush Mohammadi, Hoorieh Bashizadehfakhar, Sara Aliasghari, Zahra Gholamhoseini

**Affiliations:** ^1^Craniofacial Research Center, Tehran University of Medical Sciences, Tehran, Iran; ^2^Oral and Maxillofacial Surgery Department, School of Dentistry, Tehran University of Medical Sciences, Tehran, Iran; ^3^Department of Dentomaxillofacial Radiology, Faculty of Dentistry, Tehran University of Medical Sciences, Tehran, Iran

## Abstract

Central giant cell granuloma (CGCG) is considered a benign intraosseous lesion with a varied range of clinical features in two subtypes, including aggressive and non-aggressive lesions. This study presents a 9-year-old boy with multiple bilateral CGCG in the mandible without any systemic disease or specific syndrome. Clinical, radiographic, and histopathological findings demonstrated the aggressive lesions. It is discussed how the differential diagnosis and treatment can be determined based on the patient's age as well as the size and manner of the lesion.

## 1. Introduction

Central giant cell lesion of the jaws is a localized, benign, but sometimes aggressive osteolytic lesion [1]. Central giant cell granuloma (CGCG) is characterized by an unencapsulated proliferation of mononuclear spindle-shaped and polygonal cells with osteoclast-type multinucleated giant cell in a vascular background, with hemorrhage and hemosiderin pigment. The lesion may have a lobular architecture separated by fibrous septa with osteoid and woven bone [2].

The literature demonstrated that this lesion affects patients aged 0–85 years, with the highest prevalence in the fifth and then fourth decades of life, and a little bit shorter of 50% of all cases appear in individuals younger than 20 years [1, 3]. CGCG shows a female predilection (2 : 1), probably because of hormonal factors (pregnancy hormones and estrogen) [4, 5].

CGCGs develop twice as often frequently in the mandible than in the maxilla. However, there was no clear prevalence concerning the different regions of the jaws [1, 5, 6].

CGCGs have a wide range of clinical manifestations, from asymptomatic, slow-growing lesions without recurrence to rapidly growing, painful, and aggressive lesions with cortical plate thinning and perforation, as well as soft tissue mass and mucosal ulceration [2, 6–9].

The aim of this study is to report unusual bilateral synchronous, aggressive CGCGs in an 8-year-old male patient, which is located in ascending ramus extended to premolar area with two different treatments.

## 2. Case

A 9-year-old boy was referred to the Oral and Maxillofacial Surgery Department, Faculty of Dentistry, University of Tehran, Tehran, Iran, with the chief complaint of painful swelling on the right side of his face for the past five months. Swelling had gradually increased to its present size (as the size of an orange). No relevant medical or dental history or drug allergies have been reported.

**Figure 1:**
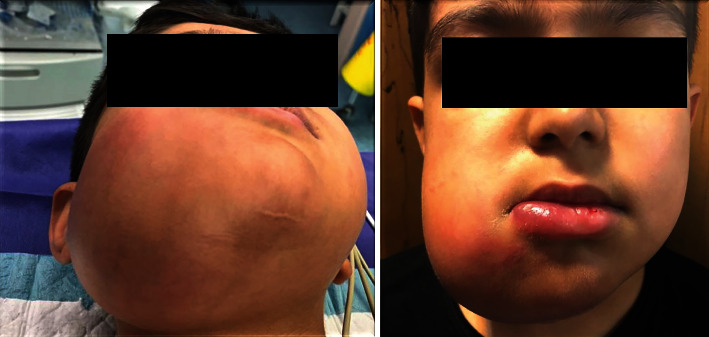
The extraoral view of the patient.

Extraoral examinations showed facial asymmetry on the right side due to a firm swelling extended from the right inferior mandibular border to the right infraorbital rim and from the right ala to the right ear lobe. Nontender expansion was obvious in the right posterior mandibular buccal and lingual cortical plates in intraoral examination (Figure 1).

**Figure 2:**
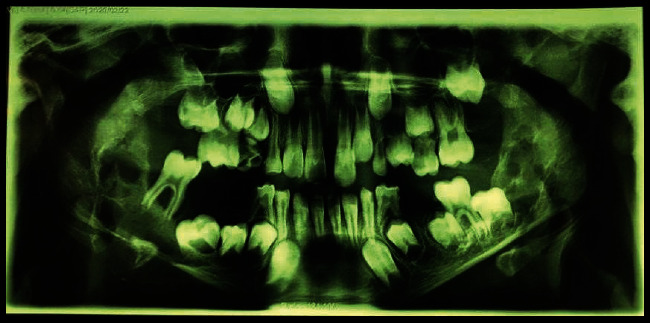
The panoramic view.

Panoramic view revealed bilateral radiolucent multilocular lesions in the primary molar area extended to the ascending ramus. The alveolar crest and inferior border of the mandible were also expanded (Figure 2).

**Figure 3:**
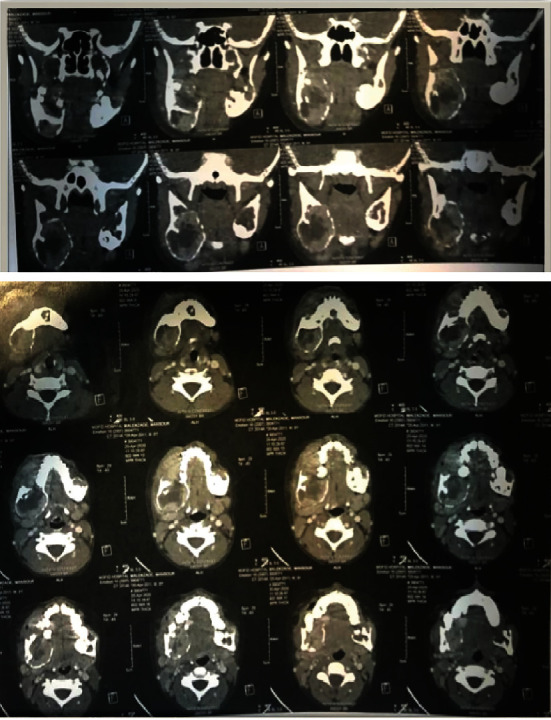
The coronal (above) and axial sections (below) of post-contrast CT.

Soft tissue window, axial, and coronal post-contrast computed tomography (CT) revealed well-defined, marked, expansile lytic lesions with enhanced borders and heterogeneous internal structure on both sides, causing buccal and lingual mandibular cortical plate expansion and thinning, as well as lingual perforation in some areas. Expansion on the right side was more noticeable (Figure 3). In serologic investigations, serum levels of calcium, phosphorus, and alkaline phosphatase were normal, vitamin D was insufficient, and parathyroid hormones (PTHs) were slightly increased (see Table 1).

**Figure 4:**
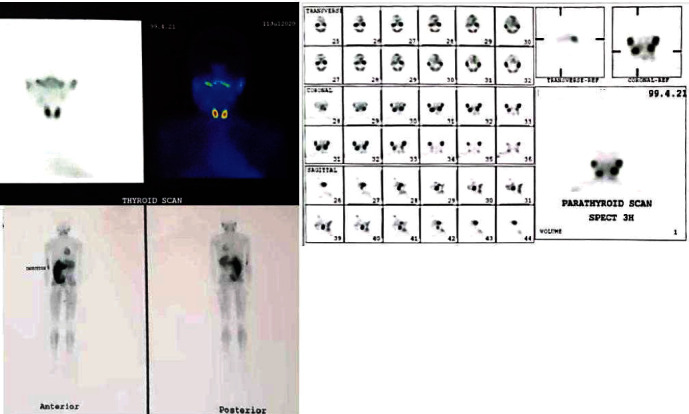
Scintigraphy of thyroid (left above), parathyroid (right above), and whole-body scan (left below).

Based on the laboratory findings, the parathyroid, thyroid, and whole-body scintigraphy were performed to rule out malignancy (Figure 4). Results showed normal thyroid and parathyroid. It was also proved that the right-sided lesion of the mandible was not at high risk for malignancy. The patient received 50,000 units vitamin D pearl weakly for one month. (In subsequent blood chemistry tests, PTH and vitamin D were found to be within the normal limits.)

**Figure 5:**
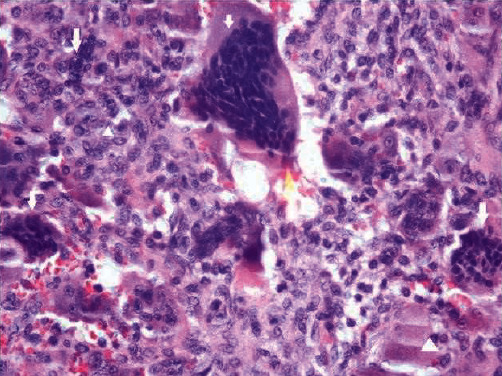
Oval and spindle mononuclear cells (arrowhead) and clusters of multinuclear giant cells (arrows).

**Figure 6:**
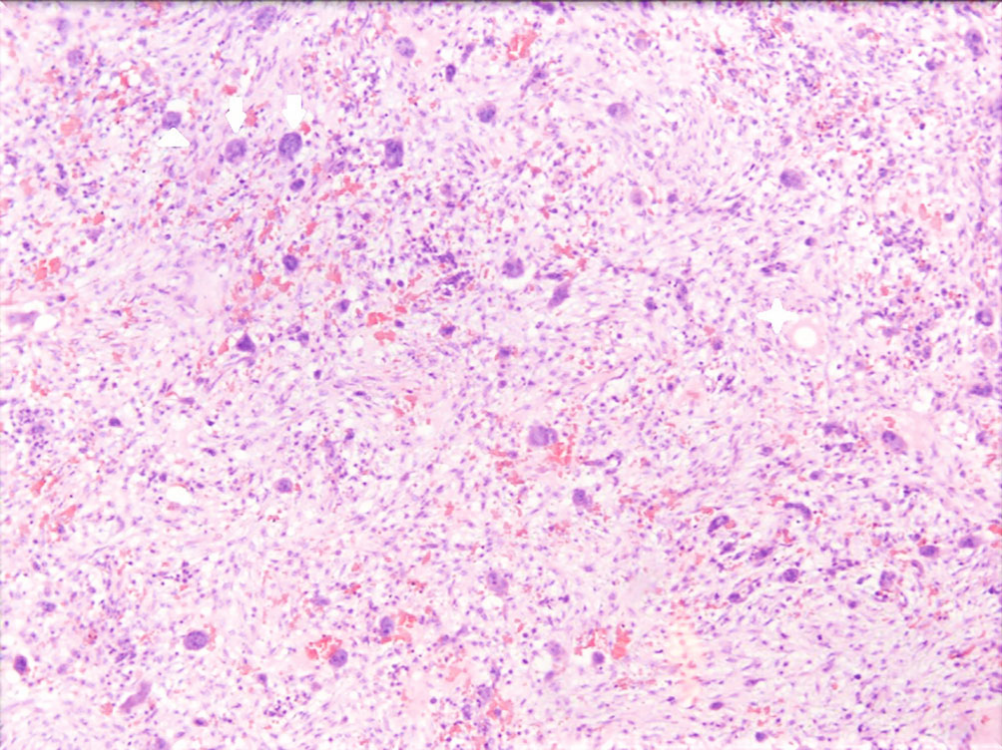
Oval and spindle mononuclear cells (arrowhead), clusters of multinuclear giant cells (arrows), and capillary (star).

An irregular, creamy-brownish soft, and bone tissue specimen measuring 4 cm × 3 cm × 1 cm was removed by incisional biopsy of the right-sided lesion. Histopathological examinations declared fibrillary connective tissue composed of stroma with small oval and spindle mononuclear cells admixed with uneven clusters of multinuclear giant cells. Small capillaries and notable mitosis were also seen. No evidence of pleomorphism was seen (Figures 5 and 6).

The numerous giant cells in varying sizes and their distribution in fibrotic connective tissue indicated an invasive giant cell lesion that, along with the pain reported, led to segmental mandibulectomy with safe margins under general anesthesia.

After surgery, the removed lesion on the right side consisted of multiple pieces of gray-brownish soft and bone tissue measuring 10 cm × 7 cm × 2 cm, along with two pieces of gray-brownish tissue measuring 1.5 cm × 1 cm × 0.5 cm that had been removed from the left side by incisional biopsy, were reevaluated histopathologically. The results revealed fragments of spongy bone trabeculae, which was extensively infiltrated by giant cell lesion, including large sheets and nests of multinucleated giant cells spread in a fibrohistiocytic stroma. Some foci of necrosis, hemorrhage, and calcification were also noted. CGCG's nature of the lesions was confirmed by these findings.

**Figure 7:**
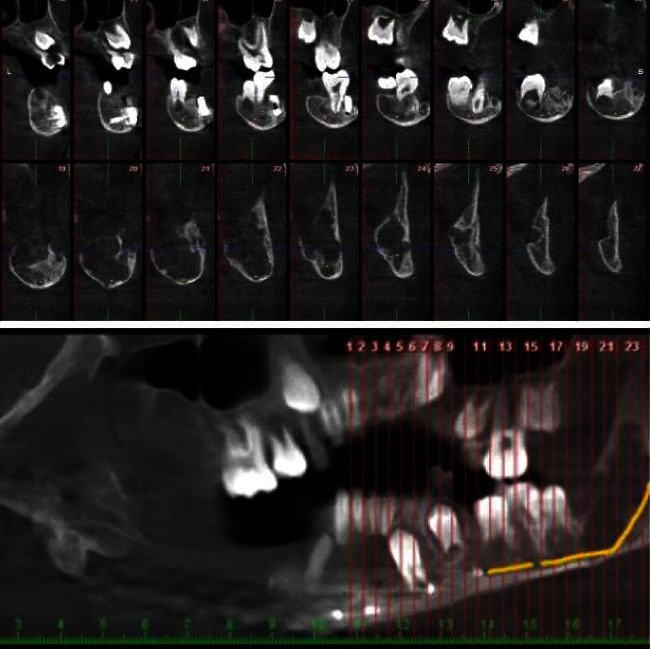
The cross-sectional images in the CBCT scan.

About five months after the first surgery, the patient complained of painful swelling on the left side. Cone-beam computed tomography (CBCT) scan of the present lesion demonstrated a well-defined corticated multilocular radiolucent lesion with the scalloped border from the posterior left mandibular body extended to the ascending ramus, leading to buccal and lingual uneven expansion and thinning (Figure 7).

Triamcinolone 45 mg along with 20 mg lidocaine HCL, and 12.5 mg epinephrine were injected into the lesion six times with three-week intervals to treat the lesion. Six months after injections, there was no progression in expansion, and the right side was reconstructed with a prosthesis.

The lesion entity and its surgical procedure were completely explained to the patient, and written informed consent was obtained for reporting the case.

## 3. Discussion

The CGCG was first defined by Jaffe in 1953 [10]. It is questionable whether the lesion is reactive or neoplastic in its nature. Jaffe considered CGCG as a reactive condition [10], whereas some authors described the lesion as a benign tumor [11–13]. The occurrence of multiple CGCGs, synchronously or metachronously, is rare. Synchronous lesions describe the simultaneous occurrence of multifocal CGCG, whereas metachronous occurrences represent recurrences due to previous incomplete surgical excision [5, 14–16]. Synchronous involvement is generally associated with syndromes or systemic disorders like brown tumor in hyperparathyroidism, Noonan syndrome, cherubism, fibrous dysplasia, ossifying fibroma, Paget's disease, or fibroosseous lesions. The simultaneous occurrence of multiple CGCGs without systemic disease or familial history is extremely rare [4, 16–19]. Multifocal CGCGs could be associated with the brown tumor of hyperparathyroidism. Microscopic features of CGCG may be indistinguishable from brown tumors. Thus, serological evaluations are crucial in determining the diagnosis. Increased levels of calcium, alkaline phosphatase, and PTH, and decreased levels of phosphorous are prominent features of hyperparathyroidism [2, 7, 20–22].

In the current patient, calcium, alkaline phosphatase, and phosphorus were within normal limits. There was a slight increase in PTH, which is justified by vitamin D insufficiency, which rules out the brown tumor from differential diagnoses. The patient was prescribed vitamin D pearls (50,000 unit vitamin D pearl weakly for one month) and reached normal serum level in subsequent tests together with PTH level. For further assessment, the patient was referred for whole-body scintigraphy. This procedure revealed no evidence of a parathyroid adenoma. Noonan syndrome is a congenital genetic disease known as autosomal dominant due to mutations in the *PTPN11* gene on chromosome 12, with a prevalence of 1 : 1000–1 : 2500 live births. Because multiple aggressive CGCs are a common feature of Noonan syndrome, it is included in the differential diagnosis in our case. The absence of symptoms, such as short stature, broad or webbed neck, low set and posteriorly angulated ears, ptosis, hypertelorism, and downward-slanting eyes in the current patient, as well as low intelligence or developmental delay, downward-slanting eyes, ptosis, and pulmonary stenosis, rules out Noonan syndrome [5, 21, 23–25].

Paget disease is a polyostotic skeletal disorder in populations over 40 years old. Paget disease may be associated with malignant tumors, including osteosarcoma, fibrosarcoma, and benign tumors, such as CGCG. Radiographically, a cotton wool appearance and an elevated level of serum alkaline phosphatase and hydroxyproline in urine are noted [17, 23, 25–27]. Therefore, Paget disease is also ruled out in our case.

Cherubism is characterized by the appearance of symmetrical, multiquadrant, multilocular, painless, and expansile radiolucent lesions in posterior sections of both jaws in a young age group, typically between the ages of 2 and 7 years. Submandibular lymph node involvement led to swelling and facial fullness in the early stages of the disorder [5, 6, 23, 25, 28–30]. Cherubism has a similar histological appearance to CCCG, it is distinguished by the presence of eosinophilic perivascular cuffing, and their absence, however, does not rule out cherubism as a diagnosis. Asymmetric and painful expansion in our patient and the absence of a similar disease in his familial history due to the autosomal dominant inheritance pattern, as well as the lack of nodular lymph nodes, can be used to rule out cherubism [30, 31].

Depending on the lesion behavior based on histological and radiographic features, different treatments from medical to surgery are considered [32]. The size, location, and manner of the lesion led to a variety of surgical procedures including local excision, curettage, resections, and even peripheral ostectomy to minimize the chances of recurrence [8, 16, 19, 33].

Medical treatment of this lesion includes intralesional steroids or corticosteroids, subcutaneous calcitonin, alpha-interferon, or a combination [3, 23, 34]. The use of intralesional corticosteroid injections as an alternative treatment was published in 1988 by Jacoway [35]. The literature shows that corticosteroid injections for 6 weeks have a direct role in reducing bone resorption based on inhibiting osteoclast formation and activity [6, 36–39]. Daily subcutaneous injections of systemic calcitonin are another effective therapy in CGCG to decrease bone resorption by allowing optimal retention of calcium in the bones, hence obviating osteoclast recruitment and maturation [6, 34, 39]. Systemic subcutaneous injections of alpha-interferon, due to its antiangiogenic function, are useful for CGCG treatment by preventing the proliferation of vascular tissue, which is common in these lesions [6, 36, 39, 40].

Previous studies have postulated the presence of pain, a size of more than 5 cm, rapid growth, tooth displacement or root resorption, and cortical plate thinning or perforations, which are reported in our case and are indicative of considering the lesion as an aggressive type. Studies have also shown that the aggressive one is more common in patients with a mean age of 10.7 years [3, 6, 41, 42].

In addition, some studies have found that aggressive variants have a higher number of giant cells, a greater surface area, and numerous mitotic activities in their histopathologic features [7, 21, 23, 41]. Others believe that lesions are invasive based on their clinical and radiographic criteria, but that the microscopic appearance is irrelevant to whether or not the lesion is invasive [2, 9, 43].

Based on clinical and radiographic criteria as well as pathologic findings, the lesion in the present case is regarded as an invasive CGCG. The young age of the patient, along with increasing pain and swelling, caused partial mandibulectomy for the lesion on the right side. Triamcinolone 45 mg was injected into the lesion on the left side six times with three-week intervals to treat the lesion due to its early stage. Up to date, no evidence of recurrence has been diagnosed.

## 4. Conclusion

CGCG should be considered as a diagnosis of a lesion with rapid growth when it comes to children. Its prompt diagnosis plays a vital role in its treatment and can help overcome the challenges. In our case, the injection of corticosteroids not only eliminated the pain but also decreased the size of the lesion. The best protocol to manage aggressive and nonaggressive lesions is yet to be determined.

## Figures and Tables

**Table 1:** Serologic investigations.

Biochemistry test	Results	Unit	Reference values
Calcium	9.8	mg/dl	8.6–10.3
Phosphorus	4.9	mg/dl	3.0–5.7
Alkaline phosphatase	586	U/l	180–1200
C-reactive protein (quantitative)	9	mg/l	<6
Parathyroid hormone	70	pg/dl	15–65
25OH VID3	11.5	ng/ml	Deficient: <10, insufficient: 10–29, and sufficient: 30–100

## Data Availability

The Data are available from the corresponding author on reasonable request.
